# IL4i1 and IDO1: Oxidases that control a tryptophan metabolic nexus in cancer

**DOI:** 10.1016/j.jbc.2023.104827

**Published:** 2023-05-23

**Authors:** Leonie Zeitler, Peter J. Murray

**Affiliations:** Max Planck Institute of Biochemistry, Martinsried, Germany

**Keywords:** amino acid, aryl hydrocarbon receptor, dendritic cell, GCN2, IDO1, IL4i1, macrophage, NRF2, SLC7A11, tryptophan

## Abstract

Regulated tryptophan metabolism by immune cells has been associated with the promotion of tolerance and poor outcomes in cancer. The main focus of research has centered on local tryptophan depletion by IDO1, an intracellular heme-dependent oxidase that converts tryptophan to formyl-kynurenine. This is the first step of a complex pathway supplying metabolites for *de novo* NAD^+^ biosynthesis, 1-carbon metabolism, and a myriad of kynurenine derivatives of which several act as agonists of the arylhydrocarbon receptor (AhR). Thus, cells that express IDO1 deplete tryptophan while generating downstream metabolites. We now know that another enzyme, the secreted L-amino acid oxidase IL4i1 also generates bioactive metabolites from tryptophan. In tumor microenvironments, IL4i1 and IDO1 have overlapping expression patterns, especially in myeloid cells, suggesting the two enzymes control a network of tryptophan-specific metabolic events. New findings about IL4i1 and IDO1 have shown that both enzymes generate a suite of metabolites that suppress oxidative cell death ferroptosis. Thus, within inflammatory environments, IL4i1 and IDO1 simultaneously control essential amino acid depletion, AhR activation, suppression of ferroptosis, and biosynthesis of key metabolic intermediates. Here, we summarize the recent advances in this field, focusing on IDO1 and IL4i1 in cancer. We speculate that while inhibition of IDO1 remains a viable adjuvant therapy for solid tumors, the overlapping effects of IL4i1 must be accounted for, as potentially both enzymes may need to be inhibited at the same time to produce positive effects in cancer therapy.

The 20 proteinogenic amino acids sustain cellular biochemistry by: (i) translation, (ii) as a source of carbon skeletons and nitrogen for energy generation and nucleotide biosynthesis, and (iii) as “shuttles” to move molecules to different cellular compartments and tasks such as the malate-aspartate shuttle or methionine as a major methyl donor for the 1-carbon-folate cycle. Several amino acids have dedicated additional functions as precursors of key signaling molecules (neurotransmitters), cofactors (heme, from glycine), or redox protective molecules such as glutathione (from glycine, cysteine, and glutamate). Because of these diverse functions, cells balance their amino acid sources by synthesis, transport, and scavenging from protein sinks and therefore constantly monitor deficiencies or excesses of all amino acids.

An additional facet of amino acid metabolism concerns the cellular and biochemical outcomes where a regulated enzyme consumes an essential amino acid and generates distinct products. In this setting, two events occur simultaneously: the essential amino acid can become limiting, triggering cellular defenses including increased amino acid transport, the reduction of translation, and activation of autophagy (and related cell protective measures) while also the products can have modulatory effects. We define “regulated” amino acid metabolism here as (i) the specific, induced expression or activity of amino acid metabolizing enzymes *via* cytokines, pathogen products, or other pathways, or (ii) the acquisition of enzyme expression not normally present in the cell type of interest. In the case of the latter, many cancer cells express amino acid metabolizing enzymes in patterns that differ from their cell of origin, presumably as a way to obtain resources to sustain growth or to evade immune control.

The main amino acid-metabolizing enzymes regulated by cytokines are arginases (ARG1 in the cytoplasm and ARG2 in mitochondria), the tryptophan oxidases IDO1 (indole, 2,3 dioxygenase), and TDO2 (tryptophan dioxygenase) and IL4i1 (Interleukin 4-induced 1, a secreted oxidase) (1). Three of these enzymes also have constitutive functions: ARG1 and TDO2 metabolize dietary arginine and tryptophan in hepatocytes, while most cells express some ARG2 to control nitrogen metabolism in mitochondria. However, within cells of the immune system, these enzymes can be highly expressed and thereby consume arginine or tryptophan within local inflammatory microenvironments while at the same time generating bioactive products that have diverse and unexpected cellular effects (1, 2). Accordingly, arginine and tryptophan metabolism intersect with many elements of immune regulation and cancer biology. In some cancer cell types, TDO2 and IDO1 expression can also be uncoupled from the regulatory signals that control enzyme expression in immune cells: We discuss this aspect briefly as a counterpoint to the immune functions of arginine and tryptophan metabolism. We note that the intersection of microbial metabolism of amino acids and especially indoles by the microbiota, with host physiology is an emerging and important topic (3, 4). However, this element of amino acid metabolism is too large in scope to be included herein.

Here, we focus on tryptophan metabolism by IDO1 and IL4i1. Summaries of the biology and clinical significance of arginase metabolism and TDO2 have been published (1, 2, 5, 6, 7) and will not be covered. Nevertheless, in a general sense, all the regulated arginine- and tryptophan-metabolizing enzymes cause similar biochemical outputs: an amino acid is consumed and a product generated. In this regard, practical and conceptual issues underline all experimental research on regulated amino acid metabolism. First, in the practical realm, quantification of the effects of amino acid metabolism is intrinsically complex as the temporal effects of depletion *versus* product generation need to be experimentally accounted for often in complex cellular environments. We also do not yet have a full understanding of the effects of cell-specific amino acid limitation, which may vary from cell to cell. Indeed, different cancer cells have substantial differences in their adaptation to malignant growth (8), while normal cells from muscle to neuron to T cell will naturally have diverse amino acid requirements linked to their specific functions. Further, we only have a rudimentary understanding of how cells detect amino acids (9) and adjust their metabolism to source the correct amounts needed.

Second, in a conceptual sense, a curiosity of regulated amino acid metabolism in the immune system *via* arginases, IDO1/TDO2 and IL4i1, is their arginine- and tryptophan-centric nature compared to other essential amino acids such as threonine or branched-chain amino acids. This specificity suggests the degradation *versus* product generation pathways from arginine and tryptophan have evolved for immune-specific tasks relative to all other amino acids. As we will discuss here, the knowledge of newly discovered biology from these pathways is rapidly advancing. Nevertheless, it is important to note that the limitation of different essential amino acids can have highly specific effects. For example, the limitation of valine specifically collapses complex one of the electron transport chain (10), and dietary valine limitation eliminates stem cells from the bone marrow (11). However, in both cases, valine amounts are controlled by experimental manipulation of the food or media rather than *via* a specific enzyme that controls valine degradation. By contrast, the biology we discuss here depends on the natural activity of endogenous enzymes that regulate amino acid limitation.

## Tryptophan metabolism by IDO1 and IL4i1

**Figure 1:**
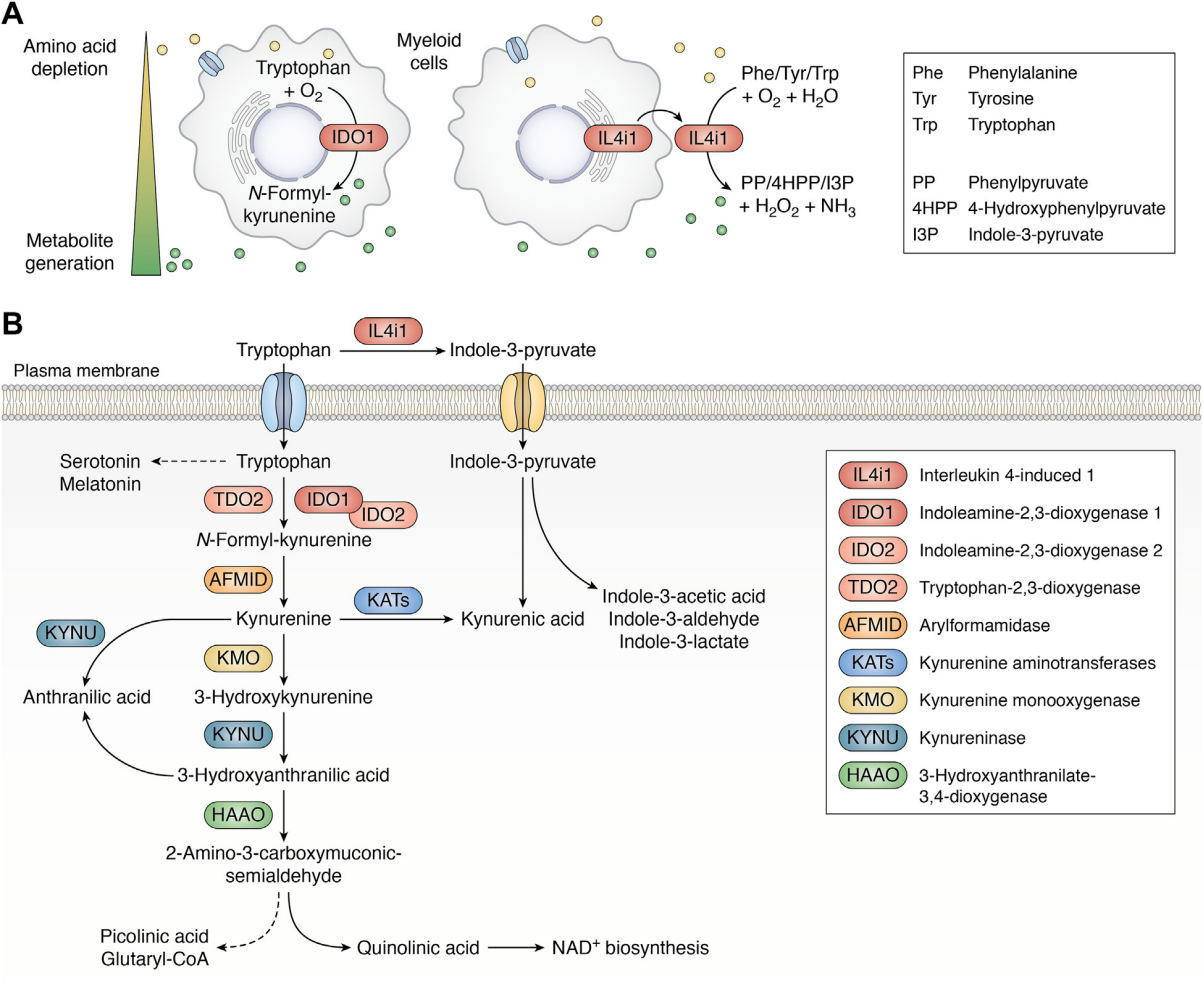
**Tryptophan and aromatic amino acid metabolism by IDO1 and IL4i1.***A*, Regulated expression of IDO1 and IL4i1 in myeloid cells can provoke a local depletion of substrate amino acids which is accompanied by the simultaneous enrichment of metabolites in the microenvironment. IDO1 is an intracellular enzyme oxidizing tryptophan (Trp) to generate N-formyl-kynurenine (N-formyl-Kyn), which subsequently gives rise to many modulatory kynurenine metabolites. IL4i1 is a secreted enzyme catalyzing the conversion of the aromatic amino acids phenylalanine (Phe), tyrosine (Tyr), and Trp into phenylpyruvate (PP), 4-hydroxyphenylpyruvate (4HPP), and indole-3-pyruvate (I3P), respectively. Additionally, the oxidative deamination reaction generates hydrogen peroxide and ammonia. *B*, Most intracellular tryptophan is metabolized *via* the Kyn pathway, which requires the conversion of tryptophan into N-formyl-kynurenine as the rate-limiting step. This reaction can be catalyzed by the enzymes IDO1, IDO2, and TDO2. The Kyn pathway gives rise to different Kyn metabolites and quinolinic acid, which is the main substrate for *de novo* NAD^+^ biosynthesis. Additionally, the Kyn pathway leads to the generation of picolinic acid and glutaryl-CoA, which can ultimately feed tryptophan carbon units into the Krebs cycle. If not metabolized *via* the Kyn pathway, intracellular tryptophan can be fed into the serotonin pathway, which can generate metabolites such as serotonin melatonin. IL4i1 acts in the extracellular space and can convert tryptophan into indole-3-pyruvate (I3P), which can subsequently be taken up by cells. The transporters involved remain to be discovered. I3P can give rise to metabolites such as kynurenic acid, indole-3-acetic acid, indole-3-aldehyde, and indole-3-lactate.

IDO1 and IL4i1 are tryptophan-metabolizing enzymes upregulated in immune cells locally depleting tryptophan (and in the case of IL4i1, other aromatic amino acids) and generating immunomodulatory metabolites in their microenvironment (Fig. 1*A*). While IDO1 is an intracellular enzyme, IL4i1 is secreted (12), suggesting that tryptophan metabolism by each enzyme occurs in a spatially separated manner. Thus, IDO1 and IL4i1 may operate simultaneously to catabolize tryptophan in the intra- and extracellular milieu.

### IDO1 and tryptophan metabolism *via* the kynurenine pathway

IDO1 catalyzes the oxidation of the tryptophan indole ring generating N-formyl-kynurenine which is the the first and rate-limiting reaction of the kynurenine (Kyn) pathway. This main tryptophan metabolic pathway generates key downstream Kyn metabolites and supplies substrates for the NAD^+^ *de novo* synthesis (13, 14, 15) (Fig. 1*B*). N-formyl-Kyn is subsequently deformylated by the constitutively expressed arylformamidase (AFMID) to generate Kyn while the formyl group is a methyl donor for 1-carbon metabolism (16). In the main branch of the Kyn pathway, Kyn is converted into 3-hydroxykynurenine (3HK) by kynurenine monooxygenase (KMO) and further into 3-hydroxyanthranilic acid (3HAA) by kynureninase (KYNU). Subsequently, 3-hydroxyanthranilate-3,4-dioxygenase (HAAO) generates 2-amino-3-carboxymuconic semialdehyde (2ACS) from 3HAA, which can spontaneously cyclize to quinolinic acid (QA), or can be converted into picolinic acid or glutaryl-CoA. Glutaryl-CoA feeds into the Krebs cycle, whereas QA is the main substrate for NAD^+^ *de novo* synthesis (13). In another branch of the Kyn pathway, Kyn can be converted to kynurenic acid (KynA) by Kyn aminotransferase (KAT) isoenzymes (15). Kyn and KynA are endogenous ligands of the aryl hydrocarbon receptor (AhR), a key ligand-activated transcription factor involved in a multitude of physiological functions including immune regulation (17), which is discussed in detail later. A notable aspect of the Kyn pathway is that KYNU and all KAT isozymes require pyridoxyl 5′-phosphate (active vitamin B6) as a cofactor (15), suggesting pyridoxyl 5′-phosphate availability in microenvironments could play a role in regulating the output of the different branches of the Kyn pathway.

Overall, by catalyzing the rate-limiting reaction of the Kyn pathway, IDO1 can modulate tryptophan availability, the emergence of Kyn metabolites, NAD^+^ biosynthesis, and fuels different metabolic pathways with tryptophan-derived carbon units. The relative expression of each enzyme of the Kyn pathway, in conjunction with substrate availability, will ultimately determine metabolic flux and the final generation of key products. Therefore, the relative expression of Kyn pathway enzymes within single cells may be highly relevant and thus appears to be an underexplored aspect of tryptophan metabolism.

Besides IDO1, two other enzymes can catalyze the first reaction of the Kyn pathway, TDO2 and IDO2, a homolog of IDO1. The genes encoding IDO1 and IDO2 are located in a tandem arrangement, suggesting they may have evolved from a common ancestral gene (18, 19). However, compared to IDO1, IDO2 exhibits a much lower catalytic activity (K_m_ 500–1000 fold lower) (18). Because of the limited information about IDO2 in the context of immunoregulation (20, 21), we do not focus on this isoenzyme in this review. TDO2 is mainly expressed in the liver where it is responsible for ∼90% of nutritional tryptophan metabolism (15). However, TDO2 expression in different cancer types (notably gliomas) is associated with tumor immune resistance (22).

### Amino acid metabolism by IL4i1

Besides IDO1, the secreted L-amino acid oxidase (LAAO) IL4i1 is another regulated tryptophan-metabolizing enzyme expressed in immune cells and associated with the regulation of immune responses (Fig. 1*A*). IL4i1 was first described in 1997 (23) as an IL-4-inducible gene in murine B cells. We now know that the main producers of IL4i1 are myeloid cells, especially macrophages and dendritic cells (DCs) (24), which we discuss extensively below. In many respects, IL4i1 is an unfortunate name as it is often confused with IL-4 and the IL-4 receptor. Moreover, the name is peripherally linked to its expression since factors other than IL-4 can control IL4i1 expression, and the protein’s function as an amino acid oxidase is not indicated. By contrast, the IL4i1 relatives in snake venoms, which provided us with a key clue about mammalian IL4i1 function (25), describe the function of the enzyme as they are called LAAOs. Nevertheless, IL4i1 is the official name of the protein and gene in mammals.

By contrast to IDO1, IL4i1 additionally metabolizes phenylalanine and tyrosine (12, 25, 26, 27). In fact, IL4i1 shows the highest substrate affinity towards phenylalanine (12, 27). Nevertheless, studies by us and others suggest that tryptophan metabolism is central for downstream cellular effects mediated by IL4i1-dependent amino acid metabolism (25, 26, 28). IL4i1 and related LAAOs, are FAD-dependent enzymes that catalyze the oxidative deamination of L-amino acids to generate α-keto acids, hydrogen peroxide (H_2_O_2_), and ammonia (29). LAAOs occur in many different organisms including not only vertebrates but also plants, bacteria, and fungi, and gained most attention as a component of snake venoms (30). Snake venom LAAOs can mediate cytotoxicity by the generation of H_2_O_2_ (31, 32, 33, 34). When we compared the enzymatic activity of recombinant mammalian IL4i1 with the Indian cobra (*Naja naja*) venom LAAO, we found that IL4i1 has a lower enzymatic activity and was not sufficient to generate toxic amounts of H_2_O_2_ (25). Besides the production of H_2_O_2_ and ammonia, IL4i1-mediated catabolism of the aromatic amino acids phenylalanine, tyrosine, and tryptophan produces the α-keto acids phenylpyruvate (PP), 4-hydroxyphenylpyruvate (4HPP), and indole-3-pyruvate (I3P), respectively (12, 25, 26). Out of these metabolites, I3P from tryptophan has the greatest downstream physiological effects including activation of the AhR and anti-oxidative properties (25). As IL4i1 is secreted, I3P generation from tryptophan occurs in the extracellular space and requires subsequent import into cells (Fig. 1*B*). However, in contrast to the well-resolved Kyn pathway, little is known about the downstream metabolism of I3P, which was recently reported to give rise to KynA, indole-3-acetic acid (I3AA), indole-3-aldehyde (I3A) and indole-3-lactate (I3L) (26).

## Expression of IDO1 and IL4i1

### IDO1 expression

In most (non-transformed) cells, baseline IDO1 expression is negligible. Instead, IDO1 expression is highly inducible. After stimulation with type 1 or type 2 interferons (IFNs), especially IFN-γ, IDO1 transcript amounts are induced thousands of fold above baseline *via* STAT1 signaling (35, 36, 37, 38). Thus, since any cell that responds to IFN-γ will also express IDO1, cell-type effects of IDO1 can be difficult to tease apart. Depending on the cell system analyzed, the effects of IDO1 on local tryptophan metabolism may be underestimated (39). A different aspect of IDO1 expression centers on cancer biology, where some tumor types (especially ovarian and endometrial tumors) express IDO1. This property can be readily observed in single-cell RNA sequencing (scRNAseq) datasets and is a foundational concept behind the use of IDO1 inhibitors in cancer therapy (40). IDO1 inhibitors may restore anti-tumor immunity, which is blocked by a tryptophan-deficient milieu resulting from IDO1^+^ myeloid cells and IDO1 expressing tumor cells. A key question about tumor-specific IDO1 expression centers on its transcriptional regulation since the involvement of IFN-independent pathways driving IDO1 expression may open up new opportunities of manipulating tryptophan metabolism in the tumor microenvironment (TME) (41, 42, 43). However, at this point, very little is understood about the extrinsic IFN-independent signals that control IDO1 expression in tumor cells. It is also possible that IDO1 is controlled by cell-intrinsic pathways that “hijack” IDO1 for enhanced tumor cell metabolism and survival. So far, these important questions remain largely unexplored but are vital to answer if we are to properly understand tryptophan metabolism in cancer.

### IL4i1 expression

IL4i1 is expressed in monocyte-derived DCs (12), macrophages residing in granulomas (24), tumor-associated macrophages (TAMs) (44, 45, 46), microglia (47), and B cells, where the enzyme was initially discovered (23, 48, 49). Furthermore, scRNAseq identified distinct macrophage and DC populations exhibiting increased IL4i1 expression (Table 1). IL4i1 expression is observed in DC subsets that were identified as migratory DCs (50, 51, 52). In addition, some of the IL4i1^+^ DC populations resemble activated DCs associated with a gene expression program linked to maturation/migration (*e.g.*, CCR7, CD40, FSCN1, and RELB) and immunoregulation (*e.g.*, CD274 (encoding PD-L1), CD200 and SOCS2), which have been termed “mature DCs enriched in immunoregulatory molecules” (mregDCs) (53). Notably, it is likely that also the DC3 population described in the study of Zilionis *et al.* (54) and the LAMP3^+^ DCs detected by Liu *et al.* (55) can be assigned to the megDC state (56). mregDCs develop from both main subsets of conventional DCs (cDCs), cDC1 and cDC2, upon capture of cell-associated antigens, which is followed by migration into the draining lymph nodes to modulate tissue and tumor-specific immunity (53). Consistent with the notion of antigen uptake by IL4i1^+^ mregDCs, also IL4i1^+^ macrophage populations identified by scRNAseq may be involved in phagocytic processes (57, 58). Mulder *et al.* (58) described an IL4i1^+^ macrophage population (IL4i1_Mac) in the TME, which in their study represented the only macrophage population expressing genes involved in phagosome maturation. This potential connection between IL4i1 expression and phagocytosis is supported by the finding that the highly phagocytic tingible body macrophages from germinal centers, which control the removal of apoptotic B cells, were found to express IL4i1 (12, 44, 57). It will be important to examine whether phagocytosis is driving the expression of IL4i1 and if IL4i1 expression has functional relevance for phagocytic processes. Furthermore, it remains to be investigated whether the IL4i1 expression in distinct macrophage and DC populations observed by scRNAseq is also reflected on protein level.

**Table 1:** IL4i1-expressing myeloid populations detected by scRNAseq

IL4i1^+^ population	Species	Context	Selection of co-expressed genes	Reference
mregDC	mouse/human	non-small-cell lung cancer; tumor and non-involved lung tissue	Aldh1a2, Ccl22, Ccr7, Cd40, Cd83, Cd200, Cd274, Fas, Fscn1, **Il4i1**, Il4ra, Pdcd1lg2, Relb, Socs2	(53)
DC3	mouse/human	non-small-cell lung cancer; tumor infiltrating myeloid cells	Ccl22, Ccr7, Cd274, Fscn1, **Il4i1**, Il12b, Marcksl1	(54)
LAMP3^+^DCs	human	nasopharyngeal carcinoma	CCL17, CCL19, CCL22, CCR7, CD40, CD80, CD83, CD200, CD274, FSCN1, IDO1, **IL4I1**, LAMP3, MARCKSL1, PDCD1LG2, SOCS2	(55)
migratory DC	mouse/human	glioblastoma	Ccr7, Cd200, Cd274, Fscn1, **Il4i1**, Marcksl1, Relb, Traf1	(51)
migratory DC	mouse	dura mater	Ccl22, Ccr7, Fscn1, **Il4i1**, Il12b, Nudt17, Socs2, Tnfrsf4	(52)
migratory cDC2	mouse	lung; infection with pneumonia virus of mice	Ccr7, Cdc42ep3, Fabp5, **Il4i1**, Relb, Spint2, Tmem176a	(50)
Il4i1^+^ cDC2	mouse	splenic DCs	Ccr7, Cd40, Cd274, Fabp5, Fas, **Il4i1**, Relb, Spint2, Tcf7	(180)
IL4i1_Mac	human	normal and tumor tissue 41 datasets from 13 tissues	CCL8, CD38, CD40, CD274, CXCL9, CXCL10, CXCL11, IDO1, **IL4I1**, LAMP3, STAT1	(58)

Examples for human and murine myeloid cell populations identified by scRNAseq showing high IL4i1 expression.

In addition to B cells, macrophages, and DCs, IL4i1 was found to be expressed in specific T cell populations including Th17 cells (59, 60) and MAIT cells (61, 62, 63). Although most T cells do not express IL4i1, it is likely that IL4i1 can accumulate in the direct environment of T cells as IL4i1 has been suggested to be secreted into the immune synapse forming between an antigen-presenting cell and a T cell (61, 64) and thus affects T cell biology even when not expressed by the T cell itself.

## Expression of IL4i1 and IDO1 in the myeloid TME

**Figure 2:**
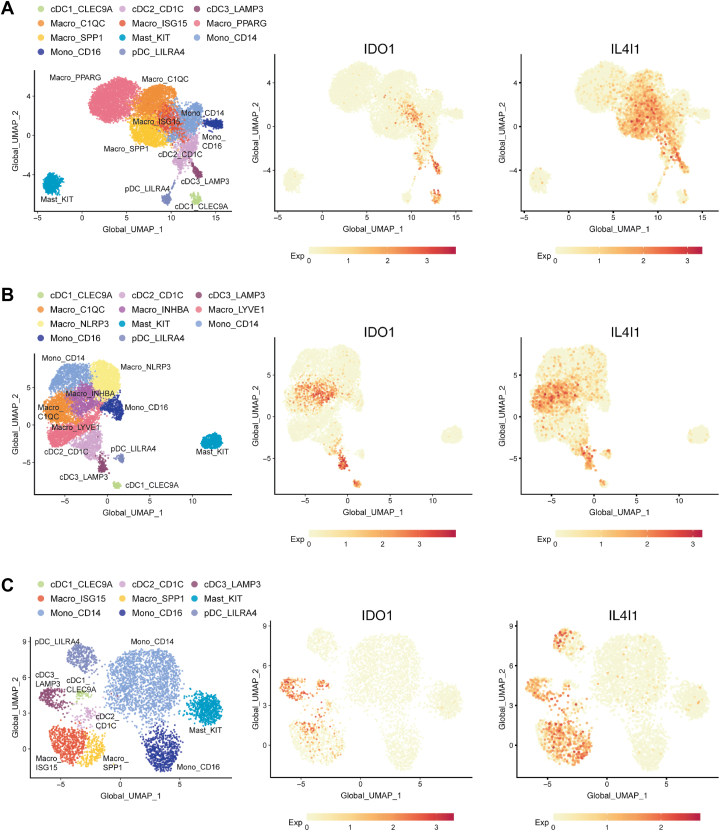
**IDO1 and IL4i1 expression in tumor-infiltrating myeloid cells.** UMAP plots showing *IDO1* and *IL4I1* expression in tumor-infiltrating myeloid cells in (*A*) lung cancer, (*B*) esophageal carcinoma, and (*C*) nasopharyngeal cancer as detected by scRNAseq in a pan-cancer study from Cheng *et al.* (65).

scRNAseq approaches revealed an unexpected element of the IL4i1 and IDO1 expression pattern in different TMEs: namely, that their expression is usually partly coincident (Fig. 2). To illustrate this, we chose three tumor types from a myeloid-enriched scRNAseq set from pretherapy patients (65). In each case, IDO1 and IL4i1 expression is enriched in myeloid cells that are LAMP3^+^ and referred to as DC3 (Table 1), which can likely be assigned to the mregDC state (56). IL4i1 expression clearly overlaps with IDO1 expression but is generally broader and occurs more frequently in macrophage populations than IDO1 expression (Fig. 2). In fact, many scRNAseq data sets show overlapping patterns of myeloid IL4i1 and IDO1 expression in the TME raising the possibility that (i) this is a universal response in the chronic inflammation of cancer and (ii) that multiple factors may control the expression of both enzymes. Determining the factors (*i.e.*, cytokines, innate immune stimuli and cell-intrinsic pathways) that control IL4i1 and IDO1 in the TME is a significant research objective because the pathways involved could be targeted to suppress expression and thus enzyme activity. The presence of different IL4i1^+^ and IDO1^+^ myeloid cells in tumor-draining lymph nodes is less understood but given that many DCs are migratory, this is an important point to consider. Many scRNAseq experiments focused on immune cells use indexing methods (*e.g.*, CITE-seq) that excludes other cell types. Indeed, a recent study of tumor stromal cells reveal high and coincident expression of IDO1 and IL4i1 (66) in non-immune cells. While these data require confirmation with proteomic and metabolomic approaches, the overlapping expression of IL4i1 and IDO1 in the TME raises a significant issue for cancer therapy approaches that target either IDO1 or IL4i1: namely, that both enzymes are generating a potentially similar aromatic amino acid metabolic milieu that may be redundant. In other words, both enzymes may have to be blocked at the same time to disrupt the pro-tumor effects of either enzyme.

## Mechanisms of immunoregulation by tryptophan metabolism

### Immunoregulatory effects of IDO1 and IL4i1

Since the first studies on IDO1 in pregnancy the main mechanistic argument for IDO1’s immunoregulatory roles has historically centered on the effects of tryptophan depletion and the generation of Kyn pathway metabolites, which can act *via* activation of the immunoregulatory aryl hydrocarbon receptor (AhR) pathway. Based on the use of first-generation IDO inhibitors, IDO1-dependent tryptophan metabolism was proposed to be required for immune tolerance in pregnancy (67) and additionally linked the catalytic activity of IDO1 to the limitation of T cell proliferation (68). These early discoveries about IDO1 need to be interpreted with considerable caution as there are doubts about the efficacy and specificity of the initial IDO1 inhibitor 1-methyl-tryptophan (36, 69) and the fact that mice lacking both IDO1 and IDO2 do not show breeding defects or any obvious autoimmune issues under normal housing (70, 71). Nevertheless, since these earlier studies, the role of IDO1 in regulating immune responses has expanded and comprises for example reduced CD8^+^ T cell proliferation (72, 73, 74), promotion of Treg differentiation (74, 75, 76), suppression of Th17 cells (77, 78), and control of graft-*versus*-host disease (79). IDO1 is also involved in immunologic tolerance in macrophages and DCs as it is required to mediate a tolerogenic macrophage phenotype in response to apoptotic cells (80), and a recent study argues that IDO1-expressing DCs can also induce IDO1 in other DC subtypes to propagate immune tolerance (81). IL4i1 is also associated with a plethora of immunoregulatory effects which comprise, for example, decreased T cell and B cell proliferation (12, 48). Moreover, IL4i1 enhances the differentiation of regulatory T cell populations while limiting the emergence of Th1, Th2, and Th17 cells (59, 60, 82) and was suggested to promote the alternatively activated M2 macrophage phenotype and IL-10 production in murine bone marrow-derived macrophages (83). The mechanisms underlying the immunoregulatory effects of IDO1 and IL4i1 likely comprise a combination of amino acid depletion and the generation of metabolites in the local environment.

### Cellular detection of amino acid limitation

Cells integrate information about the availability of amino acids for key cell fate decisions such as proliferation. Obviously, cells cannot divide without sufficient amino acids to increase cell mass for division. Therefore, the translation apparatus itself is the main amino acid sensor. Two kinases, general control nonderepressible 2 (GCN2) and mammalian target of rapamycin (mTOR), are involved in the sensing of amino acids and linked to the regulation of translation. Simplistically, while GCN2 gets activated by amino acid deprivation (84), mTOR signaling *via* the mTOR complex 1 (mTORC1) is switched off when amino acids, especially leucine and arginine, are depleted (85, 86, 87). Although the exact mechanisms of GCN2 activation are not completely understood, the sensing of increased levels of free, uncharged tRNAs (not coupled to an amino acid) is one mechanism involved. These uncharged tRNAs may bind to the histidyl-tRNA-synthetase-like domain of GCN2 and lead to conformational changes and activation of the kinase domain (88). Once activated, GCN2 phosphorylates a serine residue of the eukaryotic translation initiation factor eIF2α, which blocks the initiation of protein translation by preventing the assembly of the functional eIF2–GTP–methionyl-initiator tRNA ternary complex (89). However, while blocking most mRNA translation, the translation of specific transcripts associated with stress responses such as the transcription factors ATF4 and CHOP is increased (89).

Munn *et al.* (73) observed that tryptophan depletion by IDO1 expressed in plasmacytoid dendritic cells from tumor-draining lymph nodes suppressed T cell proliferation by GCN2 activation. In this study, GCN2-deficient T cells still proliferated under these tryptophan-limiting conditions (73). However, later studies failed to observe the rescue of T cell proliferation in GCN2-deficient T cells under conditions where amino acid availability was restricted (90, 91). A comprehensive analysis of GCN2-deficient CD4^+^ and CD8^+^ T cells showed that the limitation of tryptophan causes cell cycle arrest, independent of GCN2, although tryptophan depletion activated GCN2 signaling in the wildtype counterparts (91). Collectively, the exact role of GCN2’s amino acid detection pathway in T cells remains incompletely understood. However, it is important to consider that immunomodulatory, amino acid–depleting enzymes simultaneously generate metabolites that contribute to immunomodulatory effects. This concept is substantiated by a study showing that GCN2-dependent downregulation of the T-cell receptor (TCR) ζ-chain and resulting proliferative deficits in CD8^+^ T cells induced by IDO1 required a combination of low tryptophan levels and the presence of kynurenine metabolites (74). Moreover, we found that kynurenine can indirectly promote GCN2 signaling by competing with cysteine uptake (36). Thus, in the context of IDO1, kynurenine generation may be required to support the tryptophan starvation-induced GCN2 activation in T cells. Besides affecting T cell biology, GCN2 drives immunosuppressive macrophage and myeloid-derived suppressor cell (MDSC) function (92). In macrophages, IDO1-dependent GCN2 activation can induce a tolerogenic phenotype characterized by the production of anti-inflammatory IL-10 and the suppression of IL-12 (80). Thus, GCN2 activation by regulated amino acid metabolism may also contribute to the suppression of T-cell responses by promoting immunosuppressive myeloid cells. Overall, while many studies report IDO1-dependent GCN2 activation, it remains to be investigated if aromatic amino acid metabolism by IL4i1 activates GCN2 and whether this is involved in the immunoregulatory activities of the enzyme.

By contrast to GCN2, the mTORC1 kinase complex integrates information about sufficient nutrient availability, including amino acid sufficiency, and promotes translation and cell division. Activated mTORC1 phosphorylates the ribosomal protein p70 S6 kinase (S6K) and (hyper)phosphorylates the eukaryotic translation initiation factor eIF4E binding proteins, which leads to the release of eIF4E for the initiation of cap-dependent mRNA translation (93). Upon amino acid starvation, mTORC1 signaling is deactivated in a partial and temporal way—the extent of this effect depends on the specific amino acid that is depleted (94, 95, 96, 97, 98). Withdrawal of arginine or leucine most potently suppresses mTORC1 activity, which is linked to specific arginine and leucine binding proteins that act upstream of the complex (85). This raises the important question of how mTORC1 specifically senses the availability of other amino acids, such as tryptophan, which remains unknown.

**Figure 3:**
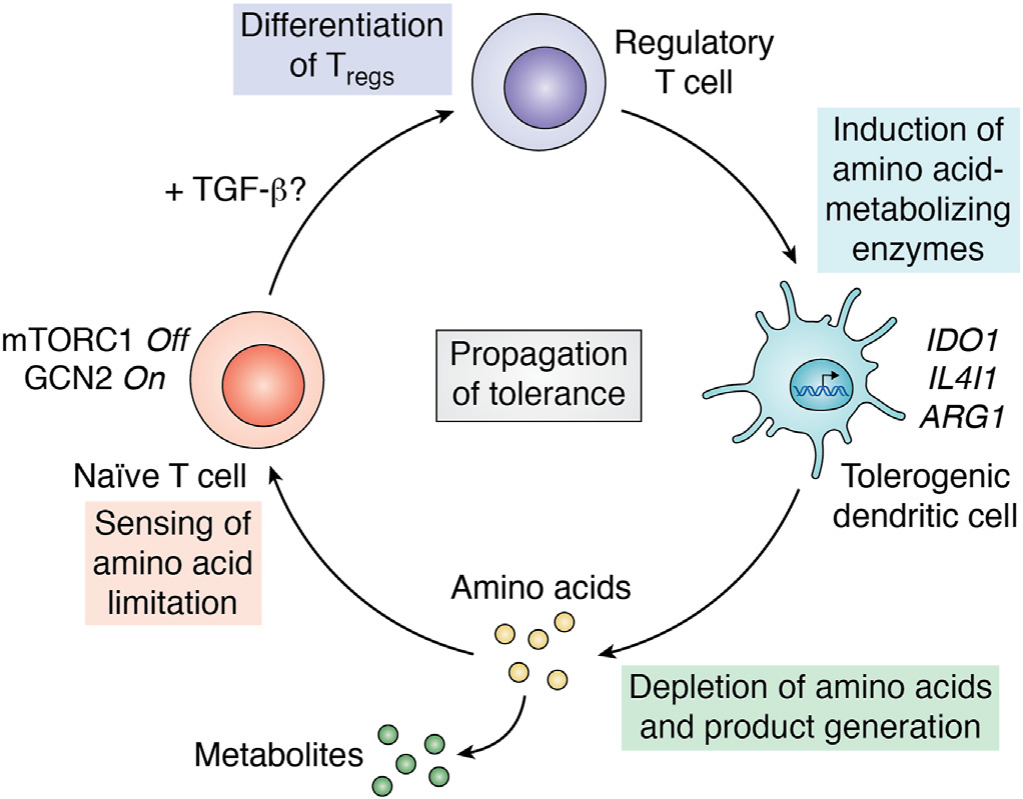
**Conceptual model of tolerance propagation by amino acid limitation.** Tregs can induce the expression of amino acid metabolizing enzymes in dendritic cells, which consequently consume their amino acid substrates in the microenvironment. The local depletion of amino acids is sensed by naïve T cells, which provokes a deactivation of mTORC1 signaling and activation of the stress kinase GCN2. These signals of amino acid limitation, likely in combination with other tolerogenic signals such as TGF-β, contribute to the differentiation of further Tregs. This cycle promotes the propagation of tolerance in a local environment.

The importance of mTORC1 in immune regulation is highlighted by the fact that rapamycin was found to be an effective immunosuppressant even before its target mTOR was discovered (99). Rapamycin blocks the G1-to S-phase transition in IL-2-stimulated T cells (100, 101) and promotes T cell anergy (102), whereas an intact mTORC1 is required for the exit of naïve T cells from quiescence and proliferation upon antigen stimulation (103). Additionally, while loss of mTOR in CD4^+^ T cells does not prevent the development of T cells, it promotes the differentiation of Foxp3^+^ Tregs and interferes with the emergence of Th1, Th2, and Th17 effector T cells (104). Inhibition of mTOR signaling *via* amino acid starvation has been linked to infectious tolerance (90), which refers to the ability of immune cells to acquire a tolerogenic phenotype that amplifies and has been best characterized in the context of transplantation (75, 105, 106). Indeed, Tregs induce the expression of amino acid–metabolizing enzymes (including IDO1 and IL4i1) in DCs *via* an unknown mechanism, which in turn limits T cell proliferation (90). Hypothetically, the tolerogenic effects of Tregs in this setting were linked to the inhibition of mTOR signaling by amino acid starvation (in synergy with TGF-β signaling) suggesting that Treg-induced amino acid consumption by myeloid cells can propagate the emergence of further regulatory T cells (90) (Fig. 3).

### The aryl hydrocarbon receptor and tryptophan metabolism

**Figure 4:**
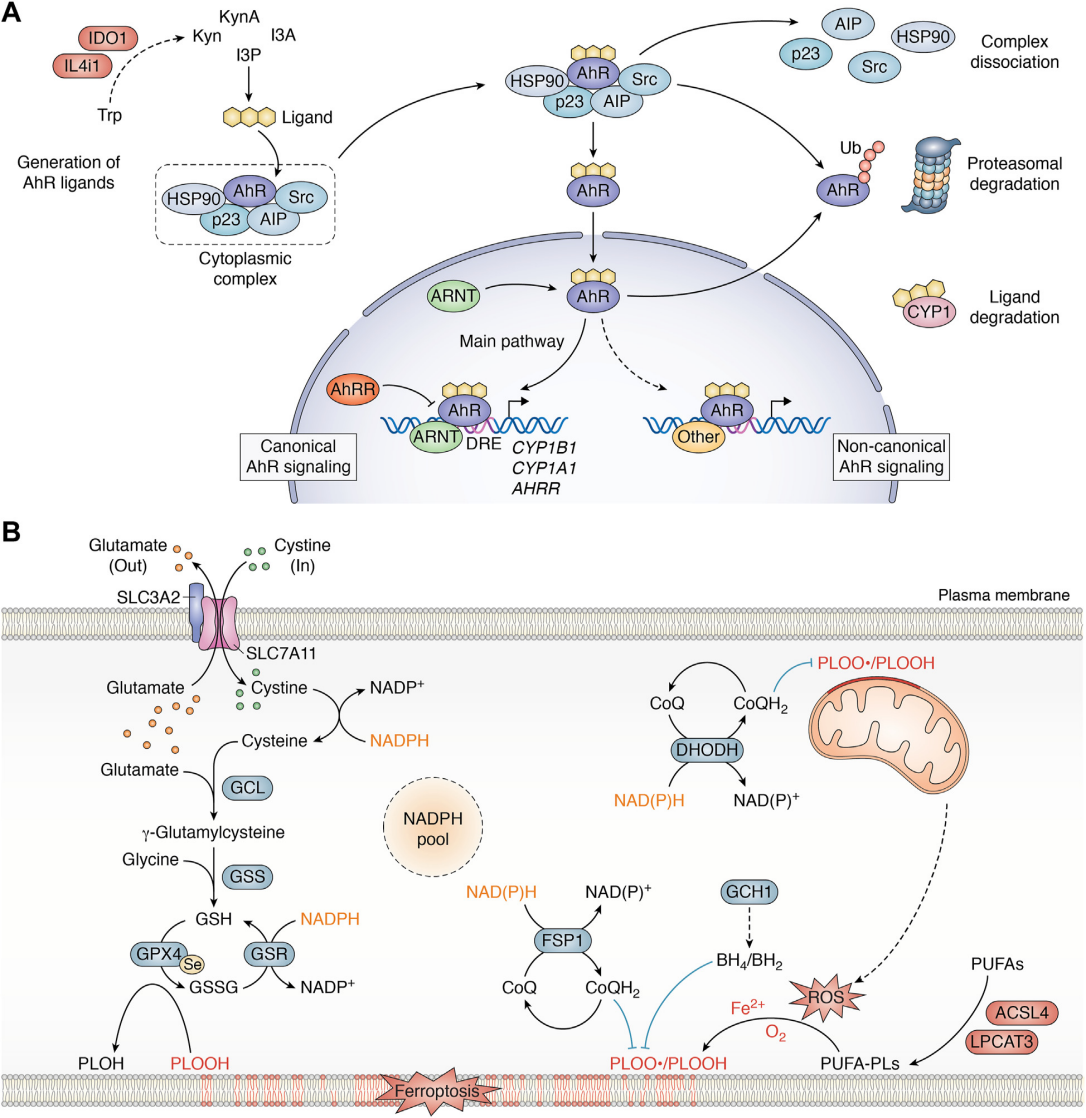
**The AhR and cell-intrinsic ferroptosis pathways.***A*, The AhR signaling pathway. Inactive AhR is bound in a cytoplasmic complex consisting of HSP90, p23, AIP, and Src. The binding of AhR ligands (*e.g.*, endogenous tryptophan metabolites generated by IL4i1 and IDO1) induces the dissociation of the cytoplasmic complex and provokes AhR translocation to the nucleus, where the receptor can interact with other proteins to induce transcription. In canonical AhR signaling AhR dimerizes with ARNT and activates gene expression at dioxin-responsive elements (DRE). Non-canonical AhR signaling involves the binding of other co-factors such as Maf or NF-κB proteins. AhR-dependent gene expression is negatively regulated by nuclear export and proteasomal degradation of AhR and by AhR target genes: The enzymes of the CYP1 family can degrade AhR ligands, while AhRR binds ARNT and thereby prevents AhR/ARNT interaction. *B*, Cell intrinsic ferroptosis-suppressive mechanisms. To prevent ferroptosis, cells have to detoxify phospholipid (PL) peroxides (PLOOH) and PL peroxyl radicals (PLOO•) that derive from the iron-dependent peroxidation of membrane PLs containing PUFAs. The main pathway detoxifying PLOOHs is the GPX4-GSH-cysteine axis (*left*). Cystine is imported into the cell *via* SLC7A11 and its co-factor SLC3A2. Subsequently cystine is reduced to cysteine by NADPH. GSH synthesis from cysteine, glutamate and glycine is catalyzed by the glutamate cysteine ligase (GCL) and the GSH synthase (GSS). GPX4 can reduce PLOOHs to non-toxic alcohols (PLOHs) while oxidizing two GSH molecules. GSH can be recovered from oxidized GSH (GSSG) by the NADPH-dependent glutathione-disulfide reductase (GSR). GPX4-independent ferroptosis suppression (*right*) can be mediated by the enzymes ferroptosis suppressor protein 1 (FSP1), dihydroorotate dehydrogenase (DHODH), and GTP cyclohydrolase 1 (GCH1). The enzymes involve the generation of ubiquinol (CoQH_2_) in the cell and mitochondrial membrane and the biosynthesis of tetrahydrobiopterin (BH_4_). These metabolites can protect cells from ferroptosis by acting as PLOO• trapping antioxidants.

The AhR is a ligand-activated transcription factor that is associated with the sensing of environmental signals including exogenous environmental toxins or endogenous ligands (107). Multiple products derived from IDO1/TDO2 and IL4i1 are AhR ligands, including Kyn, KynA, I3P, and I3A (26, 28, 108, 109, 110). Accordingly, understanding the link between the regulated tryptophan-metabolizing enzymes and AhR signaling is a key part of deconvoluting the effects of product generation by each enzyme. The AhR was initially discovered as a protein that bound the toxin 2,3,7,8-tetrachlorodibenzo-p-dioxin (111) and is therefore sometimes known as the dioxin receptor. Subsequent research revealed that AhR has multiple ligands which derive from the diet, microbiota, or endogenous generation (112). In the inactivated state (Fig. 4*A*), AhR is retained in a cytoplasmic complex comprising HSP90 (113), the co-chaperone p23 (114), the AhR-interacting protein (AIP, also XAP2) (115), and the protein kinase c-Src (116) (Fig. 4*A*). AhR ligands dissociate the cytoplasmic complex, triggering AhR translocation into the nucleus (Fig. 4*A*). There, AhR dimerizes with the AhR nuclear translocator (ARNT) to induce transcription of target genes by binding to the dioxin- or xenobiotic-responsive element (DRE or XRE) (117). To end transcription, the AhR is exported from the nucleus, ubiquitinated, and degraded by the proteasome (118). Target genes of AhR (Fig. 4*A*) include enzymes involved in xenobiotic detoxification such as cytochrome P450 family 1 (CYP1) enzymes CYP1A1 and CYP1B1 or NAD(P)H-quinone oxidoreductases. Their activity detoxifies or degrades AhR ligands, which in turn deactivates AhR signaling (119). A further feedback mechanism limiting AhR signaling is the induction of the AhR repressor (AhRR) expression, which itself is an AhR target. By binding to ARNT, AhRR limits the formation of AhR/ARNT heterodimers and thereby interferes with the transcriptional activation of AhR target genes (120). Besides the canonical signaling *via* ARNT, which likely accounts for the majority of AhR-mediated effects, AhR has also been found to interact with other transcription factors such as the retinoic acid receptor, retinoblastoma protein (Rb), c-Maf or NF-κB proteins (17).

Multiple studies have linked the activation of AhR signaling to the regulation of immune responses (reviewed by (17, 107). This includes AhR-dependent modulation of T cell biology such as the promotion of regulatory T cell subsets and Th17 development as well as the conversion of Th17 cells into IL-10-producing T regulatory type 1 (Tr1) cells (121, 122, 123, 124, 125, 126). A study showing that the tryptophan metabolite Kyn promotes the emergence of FoxP3^+^ Tregs in an AhR-dependent manner provided a first link between IDO1-mediated tryptophan metabolism and immunoregulation independent of the sensing of amino acid depletion (109). Besides effects on T cell biology, AhR signaling promotes the development of tolerogenic DCs (81, 127, 128). Notably, AhR activation can induce the expression of IDO1 (81, 127, 129, 130) and also IL4i1 has recently been suggested to be an AhR target gene (26). Thus, the IDO1- and IL4i1-dependent generation of the AhR ligands Kyn, KynA, I3P, and I3A appears to promote the maintenance and propagation of the enzymes’ own expression as shown for IDO1 (81, 127, 129, 130). This also suggests that IDO1 may induce the expression of IL4i1 and vice versa, which will require experimental validation.

We note that AhR ligands can not only derive from endogenous tryptophan metabolism but also from nutritional uptake and microbial metabolism (131). Thus, the sources and types of ligands that activate the AhR are broad and the products of IDO1/TDO2 and IL4i1 metabolism intermingle with microbial-sourced and other AhR ligands *in vivo*. Unsurprisingly, this complexity forces experimental focus on the genetic manipulation of the AhR pathway, which is possible because *Ahr* is a non-essential gene in mice.

## Regulated amino acid metabolism in cancer

As stated above, IDO1 expression is mainly controlled by the IFN pathway and can be induced in any IFN-responsive cell. However, in the TME, IDO1 is often constitutively expressed by IFN-independent mechanisms which are not well understood (41, 42, 43). Indeed, the expression of IDO1 in tumor cells was an important driver in the development of the concept that tryptophan depletion in cancer could inhibit anti-cancer immune function and ergo, IDO1 inhibitors would theoretically promote immune defense (especially T cell responses) against cancer. This subject and the apparent failures of IDO1 inhibitors in clinical trials have been extensively discussed elsewhere (40). The relevance of our discussion here is that within the milieu where tumor cells themselves acquire IDO1 expression, myeloid IDO1^+^ cells may also be present in large numbers, and we now know that IL4i1 can also contribute to tryptophan metabolic flux in the same environment. Thus, a central theme of the discussion to follow is accounting for the net effect of the different cells consuming tryptophan, and their resulting products.

Considering the expression of IL4i1 in tumor-associated myeloid cells (44, 45, 46) (Table 1), the immunoregulatory effects of IL4i1 and the involvement of other amino acid-metabolizing enzymes such as IDO1 and Arg1 in the suppression of tumor immunosurveillance (132), it is not surprising that IL4i1 gained attention as a promising target for cancer therapies over the last years. Indeed, IL4i1 is associated with reduced immune responses toward malignancies (26, 45, 46, 133) and poor survival in several cancer types including melanoma, glioma, and ovarian cancer (26, 46, 134). First evidence for the involvement of IL4i1 in the suppression of anti-tumor immune responses derived from mouse melanoma models in which IL4i1 expression provoked decreased CD8^+^ T cell responses and modulated the composition of tumor infiltrating immune cells, promoting higher numbers of immunosuppressive cell populations such as MDSCs and FoxP3^+^ Tregs while limiting tumor infiltration with CD8^+^ T cells (45, 133). These observations were confirmed in human melanoma samples with high IL4i1 expression (45, 46), and in 2020, a study from Sadik and colleagues (26) described the enrichment of immunosuppressive cells such as MDSCs and regulatory T cells as a common feature of malignancies with high IL4i1 expression. Therefore, targeting IL4i1 in cancer immunotherapy may represent a promising new strategy to restore cancer immune control.

Besides immunoregulatory effects interfering with tumor immune surveillance, IDO1 and IL4i1 may have a direct influence on controlling cancer cell biology by modulating the composition of the metabolic environment. By activating AhR signaling, Kyn and I3P were found to enhance cancer cell migration *in vitro* (26, 110, 135). Consistently, IDO1 expression was linked to increased metastasis formation in murine tumor models (136, 137, 138). In addition, tryptophan metabolism *via* the Kyn pathway was associated with resistance to oxidative stress and radiotherapy by increased *de novo* NAD^+^ synthesis (139). Recently, we and others discovered another mechanism by which tryptophan metabolism can promote cancer cell survival through IL4i1 and IDO1, namely, *via* suppression of ferroptosis (25, 36, 140, 141).

## Ferroptosis suppression by IDO1 and IL4i1

### Ferroptosis

Ferroptosis is a form of oxidative, iron-dependent cell death characterized by the uncontrolled spreading of membrane lipid peroxidation which finally provokes the loss of cell membrane integrity, a late and essential step in ferroptotic cell death (142, 143). The chemical basis of lipid peroxidation occurring within the context of ferroptosis has been extensively reviewed (144): Reactive oxygen species (ROS) that are constantly produced during cellular metabolism can initiate the formation of free radicals from phospholipids (PLs) containing polyunsaturated fatty acids (PUFAs). These radicals can subsequently react with molecular oxygen to form PL peroxyl (PLOO•) radicals (Fig. 4*B*). In turn, PLOO• radicals propagate the generation of further PUFA radicals, while free iron promotes a Fenton-like reaction to produce additional reactive radicals from the emerging PL peroxides (PLOOH). This provokes an avalanche-like propagation of lipid peroxidation, which can finally disrupt the integrity of cell and/or organelle membranes (143, 145). Acyl-CoA Synthetase Long-Chain Family Member 4 and lysophosphatidylcholine acyltransferase 3, two enzymes involved in the incorporation of PUFAs into membrane phospholipids, sensitize cells to ferroptotic death (146, 147). To suppress ferroptosis, cells depend mechanisms detoxifying membrane PLOOHs and the highly reactive PLOO• radicals. Cell intrinsic ferroptosis protection involves the glutathione peroxidase 4 (GPX4)-glutathione (GSH)-cysteine axis and other, GPX4-independent mechanisms (Fig. 4*B*). As these pathways have been described by many comprehensive reviews (140, 143, 145, 148), we will not go into much detail here. In brief, the GPX4-GSH-cysteine axis is centered on glutathione (GSH), the most abundant intracellular antioxidant (149). GSH consists of three amino acids, glutamate (linked *via* the γ-carboxyl group), cysteine, and glycine, out of which cysteine is the rate limiting substrate (140, 150, 151). Thus, cells require sufficient cysteine supply, which mainly derives from the import of cystine *via* the transporter SLC7A11 (together with its co-factor SLC3A2) or from endogenous synthesis via the transsulfuration pathway, to maintain the intracellular generation of GSH (152, 153, 154, 155). GPX4, the main PLOOH decomposing enzyme, can oxidize two GSH molecules to reduce and thereby detoxify the PLOOHs protecting cells from ferroptotic death (156, 157, 158). Other factors that act independent of GPX4 (Fig. 4*B*) comprise, for example, ferroptosis suppressor protein 1 and dihydroorotate dehydrogenase, two enzymes catalyzing the reduction of ubiquinone (CoQ) to ubiquinol (CoQH_2_) in the cell and mitochondrial inner membrane, respectively, which in turn can act as an antioxidant trapping PLOO• radicals (159, 160, 161). In a similar manner, GTP cyclohydrolase 1 catalyzes the rate-limiting reaction in the synthesis of tetrahydrobiopterin (BH_4_), which likewise protects lipid membranes by acting as an antioxidant (162, 163). Thus, the enzymatic decomposition of PLOOHs *via* GPX4 and the trapping of PLOO• radicals confers cell intrinsic protection from ferroptosis. We recently discovered that IL4i1 and IDO1 can promote cell extrinsic resistance to ferroptosis by metabolizing tryptophan and generating suppressive metabolites in their micro milieu (25, 36), which may not only protect the IDO1- and IL4i1-expressing cells but also increase ferroptosis resistance in surrounding cells. This provides a novel link between IL4i1- and IDO1-positive TMEs and cancer cell survival.

### IL4i1 and IDO1 control ferroptosis by overlapping mechanisms

**Figure 5:**
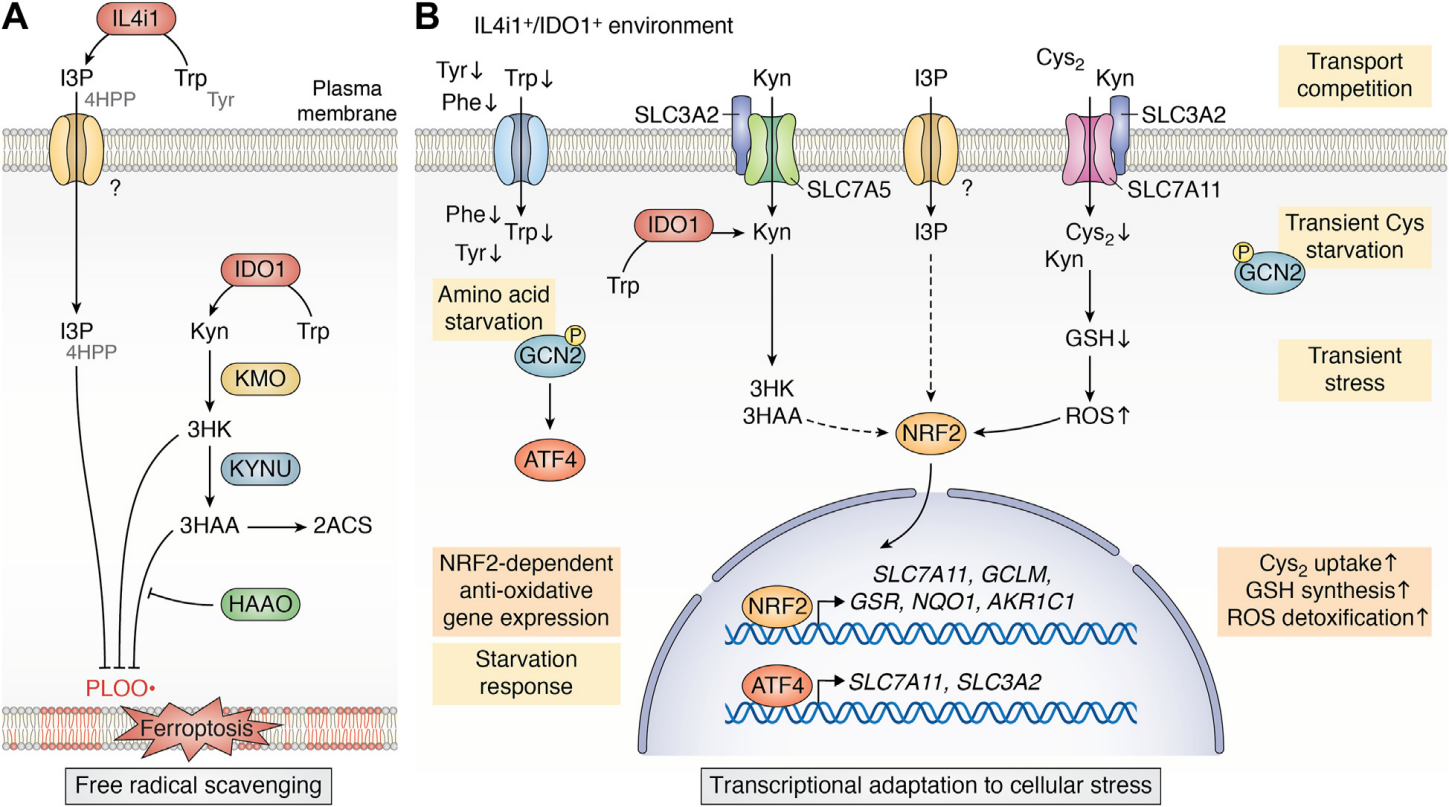
**Mechanisms of ferroptosis suppression by IL4i1 and IDO1.** Ferroptosis suppression by IL4i1 and IDO1 involves (*A*) free radical scavenging and (*B*) the transcriptional adaptation to cellular stress. *A*, The aromatic amino acid metabolites 3HK, 3HAA, I3P, and to a lesser extent 4HPP deriving from tryptophan and tyrosine, protect cells from ferroptosis by scavenging free radicals. Enzymes involved in the generation of these metabolites suppress ferroptosis, while HAAO, the 3HAA decomposing enzyme interferes with the protective effect of the Kyn metabolites. *B*, Activation of the anti-oxidative NRF2 pathway and the GCN2-ATF4 axis promote ferroptosis protection by inducing the expression of genes involved in cystine (Cys2) uptake, GSH synthesis, and the detoxification of reactive oxygen species (ROS). IL4i1 and IDO1 deplete aromatic amino acids (AA) in the microenvironment. AA starvation activates the GCN2-ATF4 axis. In addition, extracellular Kyn is transported into cells *via* SLC7A11, which is competing with Cys2 import. Transient cysteine starvation thereby contributes to the GCN2 activation. The temporary decrease of intracellular cysteine reduces GSH production and increases ROS, which in turn activates NRF2-dependent transcription. Moreover, I3P, 3HK, and 3HAA also activate the NRF2 pathway.

Amino acid metabolism by IL4i1 and IDO1 confers cell protection from ferroptosis by several mechanisms which can be subdivided into (i) the scavenging of free radicals *via* the generation of specific amino acid metabolites and (ii) the induction of stress-protective transcriptional programs (Fig. 5). Notably, these mechanisms are temporally distinct as radical scavenging can occur within seconds while the activation of protective gene expression takes hours. Nevertheless, this does not exclude that both processes act simultaneously in an environment in which IL4i1 and IDO1 are constantly expressed.

The α-keto acid I3P generated by IL4i1 from tryptophan and to a lesser extent 4HPP deriving from tyrosine are able to scavenge free radicals (25) (Fig. 5*A*), which upon uptake into cells may directly interfere with ferroptosis by trapping of the PLOO• radicals as previously observed in the context of ferroptosis suppression by CoQH_2_ and BH_4_ (159, 160, 161, 162, 163). Likewise, the Kyn pathway metabolites 3HK and its downstream metabolite 3HAA, requiring the rate-limiting reaction catalyzed by IDO1 (or TDO2) for their generation (Fig. 1*B*), can act as antioxidants. 3HAA has a higher scavenging potential at low concentrations than 3HK (36) and was specifically shown to better eliminate peroxidized phospholipids (141). Consistently, expression of KYNU, the 3HAA-generating enzyme, promotes IDO1-dependent ferroptosis protection while expression of HAAO, the 3HAA decomposing enzyme, promotes cell susceptibility to ferroptosis and interferes with the protective effect of 3HAA (36, 141). This indicates the relevance of the relative expression of different Kyn pathway enzymes for IDO1-dependent ferroptosis protection.

Besides generating metabolites that have anti-oxidant function, amino acid metabolism by IL4i1 and IDO1 activates different pathways of cellular adaptation to stress by remodeling the metabolite composition in the microenvironment (Fig. 5*B*). These mechanisms comprise the GCN2-dependent starvation response increasing ATF4-dependent transcription and the activation of NRF2 signaling, which is the main pathway involved in the sensing and protection of cells from oxidative stress (164, 165). IL4i1 and IDO1 activity decreases the amount of available tryptophan (and in the case of IL4i1 also phenylalanine and tyrosine) while provoking an enrichment of downstream amino acid metabolites in the microenvironment. As IL4i1 is a secreted enzyme, the enzymatic activity directly affects the extracellular milieu (26). Moreover, although IDO1 is located inside cells, Kyn and other Kyn pathway metabolites are released from IDO1-expressing cells, providing an extracellular supply of IDO1-dependent metabolites, which can subsequently be imported by non-IDO1 expressing cells (36, 166). Notably, we found that supplementation of extracellular Kyn partly protects cells from ferroptosis by provoking the activation of NRF2-dependent transcription but unexpectedly also the GCN2-dependent ATF4 activation despite normal levels of tryptophan in our experimental conditions (36). A clue for the underlying mechanisms for NRF2 and GCN2 activation by Kyn came from considering Kyn import in activated T cells, which was shown to be mediated by SLC7A5 forming a complex with SLC3A2 (167). Another member of the SLC7 family complexing with SLC3A2 is the cystine transporter, SLC7A11, which is a central component of the ferroptosis suppressive pathways as described above (Fig. 4*B*). Because SLC7A5 and SLC7A11 are structurally similar, we reasoned that Kyn could be imported by both transporters. Indeed, we found Kyn was rapidly imported by SLC7A11, and thus transiently competed with the uptake of extracellular cystine (36). We suggest that the transient cystine starvation provokes the Kyn-induced activation of the GCN2–ATF4 axis which, in the context of IDO1-dependent tryptophan metabolism, would add to the well-established activation of the same axis *via* tryptophan depletion (36, 73, 80, 91). Moreover, transient cystine starvation may cause a decrease in intracellular GSH production which could explain the activation of the NRF2 pathway due to a transient increase of oxidative stress. Importantly, SLC7A11 is a target gene of both ATF4 and NRF2 (168), and accordingly, we observed a massive upregulation of SLC7A11 in Kyn-treated cells. Thus, despite the competition with cystine import, steady exposure of cells to Kyn, which we assume would be the case in an IDO1^+^ TME, provokes increased uptake of cystine *via* a strong increase of SLC7A11 expression (36). Notably, a study by Procaccini *et al.* (169) reported an NRF2-dependent upregulation of SLC7A11 in Tregs under conditions of “pseudo-starvation” comprising, for example, mTOR inhibition, which was required for the induction of their regulatory phenotype and suppressive functions. This raises the question of whether SLC7A11 upregulation may be a further mechanism by which Kyn contributes to Treg differentiation apart from AhR activation. Besides the transcription of SLC7A11, NRF2 induces the transcription of a plethora of genes involved in the protection from oxidative damage including genes involved in GSH synthesis and homeostasis, such as *GCLM*, *GCLC*, and *GSR* (165) or the AKR1C family enzymes (170) which are involved in the decomposition of toxic lipid aldehydes (171) and were linked to increased ferroptosis resistance (152). In addition to Kyn, its derivatives 3HK and 3HAA are able to activate NRF2 signaling as well as I3P and to a lesser extent also 4HPP generated by IL4i1 (25, 36). So far, we do not understand how these metabolites activate NRF2 (Fig. 5*B*), which may be due to transient oxidative stress but could also result from other mechanisms such as alkylation of the NRF2 regulating protein KEAP1 as observed for the NRF2-activating metabolite itaconate (172). Other important, missing pieces of information are the respective transporters for each of the metabolites, which would also help to understand whether all cell types can import the protective metabolites.

**Figure 6:**
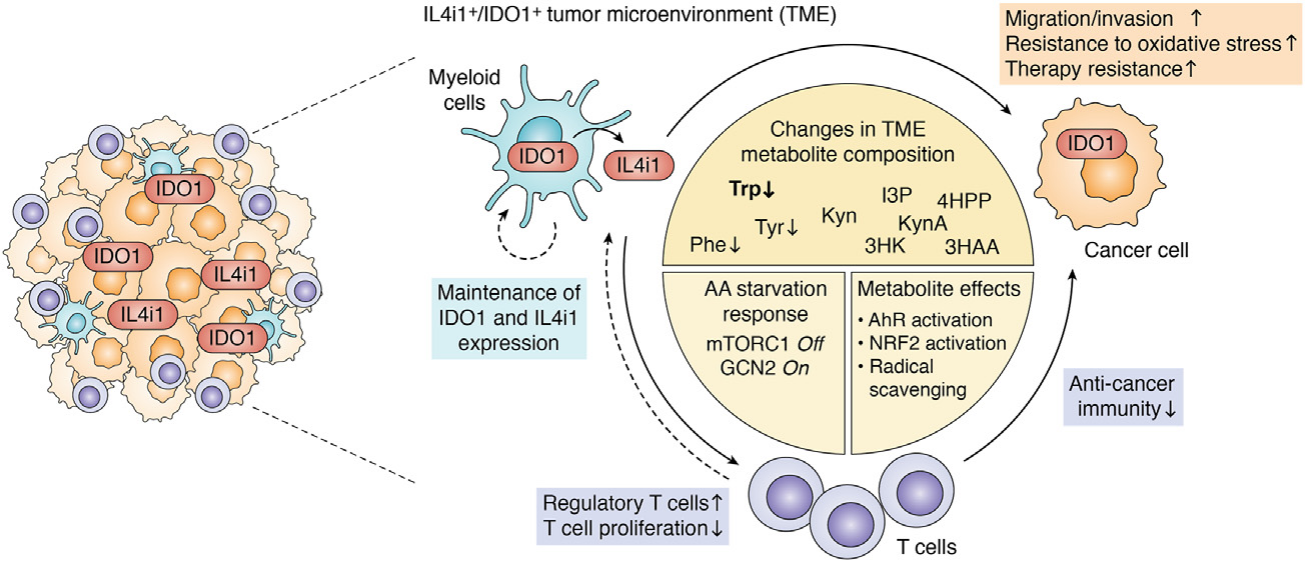
**Model of tumor-promoting mechanisms by IL4i1 and IDO1 expression in the TME.** IL4i1 and IDO1 expression by myeloid cells in the TME and the case of IDO1 also by cancer cells change the local metabolite composition by depletion of amino acid (AA) substrates and the emergence of modulatory downstream products. This can provoke an AA starvation response involving the deactivation of mTORC1 signaling and the activation of the GCN2 stress kinase. Additionally, the generated metabolites can activate AhR- and NRF2-dependent transcriptional programs and scavenge free radicals. In the immune cell compartment, this may promote Treg differentiation and function (AA starvation response, AhR activation, NRF2-dependent upregulation SLC7A11) and interfere with effector T cell proliferation (AA starvation response). A shift towards Treg-driven immune responses can interfere with anti-cancer immunity and promote the expression of IDO1 and IL4i1 in myeloid cells. In addition, due to their potential transcriptional regulation through AhR, IDO1, and IL4i1 may maintain their own expression in the myeloid immune cell compartment by generating AhR agonists. The changes in the metabolite composition may also promote tumor cell migration and invasion (AhR activation) and enhance tumor cell resistance towards intrinsic oxidative stress and stress induced by cancer therapies (NRF2 activation, radical scavenging).

Taken together, IDO1 and IL4i1 can protect cells from ferroptosis by generating a microenvironment that comprises radical scavenging amino acid metabolites and provokes an adaptation to oxidative stress by activating NRF2 and the GCN2–ATF4 axis (Fig. 5). Notably, AhR signaling seems to be dispensable for the protective effects as loss of *AHR* does not affect the protective effects of Kyn, 3HK or 3HAA (36). Furthermore, KynA, a known AhR ligand (26, 108) that can be found as a downstream metabolite of both, IDO1 and IL4i1 tryptophan metabolism (Fig. 1*B*), does not show any anti-ferroptotic activity (36). Most important, however, is the concept that one cell can generate tryptophan metabolites that control the physiology of neighboring cells. In this model (Fig. 6), a clinically relevant system would be a tumor-associated myeloid cell that generates Kyn pathway products and I3P, which then control the redox balance in a stressed cancer cell. Tumor cells with non-genetic, acquired drug resistance, so-called persister cells adapt their metabolism and frequently alter the expression of redox regulatory genes and proteins resulting in a strong dependence on ferroptosis suppressive pathways (173, 174, 175). Thus, a stressed cancer cell (*e.g.*, in chemotherapy) may be protected by the local presence of anti-ferroptotic tryptophan metabolites. Notably, the possibility of protective cell-cell communication between persister cells and myeloid cells has yet to be experimentally tackled.

## Experimental deconvolution of the IL4i1 and IDO pathways

As we have detailed so far, IL4i1 and IDO1 expression in the tumor microenvironment by myeloid cells (and in the case of IDO1 also by cancer cells) promote tumor progression by limiting tumor-specific immune responses but also by directly affecting tumor cell biology and conferring cancer cell resistance to oxidative stress (Fig. 6). Mechanistically, IL4i1 and IDO1 use tryptophan to generate I3P or kynurenines, each of which has multiple cell-intrinsic and -extrinsic effects on cell viability, metabolic signaling, and adaptive pathways. How then can we assign the relative importance of one pathway *versus* the other in a complex inflammatory environment? Hypothetically, in the case of IL4i1 and IDO1 coincident expression, tryptophan (for IDO1 and IL4i1), tyrosine, and phenylalanine (for IL4i1) will be depleted inside (IDO1) and outside cells (IL4i1); this will trigger the amino acid starvation response in a cellular milieu that lacks sufficient perfusion. At the same time, I3P, 4HPP and Kyn pathway metabolites scavenge ROS, activate the NRF2 anti-ferroptosis pathways, and activate the Ahr. Also at the same time, changes in metabolism occur *via* NAD^+^ biosynthesis, 1-carbon metabolism, and other pathways linked to tryptophan and tyrosine metabolism. Therefore, accounting for the different effects of each of these downstream pathways is a daunting challenge for quantitative cellular biochemistry and cell biology that must be solved by complementary approaches.

An informative (but simpler) comparator is how Arg1 controls T-cell responses in a complex inflammatory environment. In this example, the activation of Arg1 expression in macrophages triggers arginine hydrolysis and the production of ornithine and urea, each of which could contribute to different downstream physiological effects, which is reflected in a spectrum of pathological effects *in vivo*, from negligible or no phenotypes (asthma, *Trichuris muris* infection), to partial effects (cutaneous wound healing) to lethal inflammation (schistosomiasis worm egg deposition in the liver) (176, 177, 178). To understand the mechanistic basis of these effects, we used reduced complexity *in vitro* systems that combine genetics (*i.e.*, where macrophages are deficient in Arg1) and metabolic manipulation such as adding back precise amounts of arginine or ornithine (179). When viewed *in toto*, the final conclusion was that depletion of arginine was the key pathway Arg1^+^ macrophages use to control inflammatory responses, while ornithine and urea were dispensable. However, the effects of Arg1 are simpler than either IL4i1 or IDO1 because Arg1 controls cell function *via* arginine depletion while IDO1 and IL4i1 deplete tryptophan, generate AhR ligands and control ferroptosis.

The deconvolution of the downstream effects of IL4i1 and IDO1, therefore, requires genetic and pharmacologic manipulation of the enzymes in *in vivo* settings. This can provide valuable clues about dominant pathways, for example, by detection of the known metabolites which can inform about hierarchical metabolic pathways that are used *in vivo*.

## Double hits: IDO1 and IL4i1 and their clinical application

IL4i1 and IDO1 have an overlapping expression in inflammatory microenvironments comprising infiltrating myeloid cells and constitutive expression in some malignant cells. Therefore, an implication of the recently uncovered regulatory and metabolic pathways controlled by IL4i1 and IDO1 is that inhibition of one enzyme in cancer therapy may be insufficient to block ferroptosis suppression, AhR activation, tryptophan depletion or downstream metabolic supply *via* 1-carbon and NAD metabolism. IDO1 inhibitors were developed and tested in numerous cancer clinical settings without knowledge of the overlapping pathways regulated by IL4i1, and without knowing that both IDO1 and IL4i1 control redox stress responses. Furthermore, the fact that IDO1 and IL4i1 can both generate AhR ligands and deplete tryptophan suggests cross-talk between the two enzymes may be extensive, especially as both enzymes appear to be transcriptionally regulated by AhR to some extent (26, 81, 130). This raises the question of whether IDO1 regulates IL4i1 *via* product generation and vice versa. The primary focus of IDO1 inhibitor activity was to locally restore tryptophan availability to create a permissive environment for anti-tumor T cell proliferation and function, which we now know is only one regulatory pathway activated by regulated tryptophan metabolism. *Inter alia*, we suggest that an IDO1 inhibitor strategy may be more useful if combined with an IL4i1 inhibitor, which may be especially useful as IL4i1 is secreted and thus active in the extracellular milieu and easier (in theory) to inhibit *via* small molecules. A final point related to this concept is the necessity for precise knowledge about IDO1 and IL4i1 expression; as high-quality anti-human IDO1 and IL4i1 antibodies are available, tissue staining for the cell types and approximate amounts of either enzyme in a cancer biopsy is imperative to guide inhibitor use.

## Conclusion and open questions

New developments in tryptophan metabolism have provided opportunities to understand and exploit this complex pathway, especially in the context of cancer. The overlapping expression of IL4i1 and IDO1 especially in myeloid cells populating inflammatory microenvironments suggests that the enzymes are functionally redundant (*i.e.*, they both generate anti-ferroptotic and AhR-activating metabolites, they both deplete tryptophan) to some extent; in this case, inhibitors of both IL4i1 and IDO1 may be necessary to interrupt the three main biochemical outputs: tryptophan depletion, AhR activation, and ferroptosis suppression, all of which conceivably operate within the same space and temporal frame. Therefore, genetic and pharmacologic experiments will be essential to understand the relative effects of IL4i1 *versus* IDO1 in a given experimental setting; this is especially significant given the limited effects of IDO1 inhibitors in cancer, which were used without (i) understanding IL4i1 was likely expressed in same environments and (ii) that regulation of ferroptosis was an unknown physiological output from both enzymes.

In a shorter timescale, we have three key questions that need to be answered to move this field forward: (1) Can an experimental system be devised to quantitatively track the export and uptake of the different tryptophan metabolites in a one- cell or 2-cell system? For example, what is the simplest system to dissect the metabolic communication between one cell generating IDO1 or IL4i1 and another cell receiving the signal? (2) Which transporters are involved in the import and export of Kyn pathway metabolites and I3P? (3) Can a simple experimental system be devised to accurately measure the relative effects of the different pathways activated by IDO1 and IL4i1 metabolites? This seems possible since the genes encoding AhR, NRF2, and GCN2 are non-essential in many cells, and many points of the ferroptosis, AhR, and GCN2 pathways can be perturbed with on-target drugs.

## Conflict of interest

PJM is on the scientific advisory boards of Palleon Pharmaceuticals and ImCheck Pharma, neither of which have activities or interests related to this manuscript.
